# Design and Analysis of a Lightweight Context Fusion CNN Scheme for Crowd Counting

**DOI:** 10.3390/s19092013

**Published:** 2019-04-29

**Authors:** Yang Yu, Jifeng Huang, Wen Du, Naixue Xiong

**Affiliations:** 1 College of Information, Mechanical and Electrical Engineering, Shanghai Normal University, Shanghai 201418, China; 1000441792@smail.shnu.edu.cn; 2 DS Information Technology Co., Ltd., Shanghai 200032, China; duwen@dscomm.com.cn; 3 Department of Mathematics and Computer Science, Northeastern State University, Tahlequah, OK 74464, USA; xiong31@nsuok.edu

**Keywords:** crowd counting, convolutional neural networks, deep learning, computer vision

## Abstract

Crowd counting, which is widely used in disaster management, traffic monitoring, and other fields of urban security, is a challenging task that is attracting increasing interest from researchers. For better accuracy, most methods have attempted to handle the scale variation explicitly. which results in huge scale changes of the object size. However, earlier methods based on convolutional neural networks (CNN) have focused primarily on improving accuracy while ignoring the complexity of the model. This paper proposes a novel method based on a lightweight CNN-based network for estimating crowd counting and generating density maps under resource constraints. The network is composed of three components: a basic feature extractor (BFE), a stacked à trous convolution module (SACM), and a context fusion module (CFM). The BFE encodes basic feature information with reduced spatial resolution for further refining. Various pieces of contextual information are generated through a short pipeline in SACM. To generate a context fusion density map, CFM distills feature maps from the above components. The whole network is trained in an end-to-end fashion and uses a compression factor to restrict its size. Experiments on three highly-challenging datasets demonstrate that the proposed method delivers attractive performance.

## 1. Introduction

Automatic crowd analysis is one of the more promising solutions for urban security services. Given the increasing attention of city managers to security issues, researchers have attempted to analyze issues related to various foundational aspects, such as crowd counting (estimating the head count) [1,2], density estimation [3,4], behavior analysis [5], pedestrian tracking [6], and anomaly detection [7]. However, because of the presence of various complexities in a real scene, these techniques are far from optimal. In this paper, we further explore the task of estimating the crowd count from a single image under resource constraints.

In addition to the common challenges of messy scenes and changeable light, large variations in scale are a key factor limiting the accuracy of most proposed methods for crowd counting. Depending on the camera perspective or the real gaps between heads, areas with heads range from hundreds of pixels to a few pixels. Several CNN-based models [8,9,10,11] that are adaptive to multi-scale blobs have been proposed, and the general trend has been to make deeper and more complicated networks for higher accuracy. In the recent literature, adaption to different density domains has become a first-order question, with less attention paid to an appropriately right-sized network.

The size of these improved networks is expanding because of the special components of multi-scale or contextual features. On the other hand, some patch-based networks [11,12] fuse multi-level contextual information by cropping the original image into several patches or rescaling the input image several times. While these methods restrict the size of networks, the computation in the whole feedforward process is still increased several fold. Overall, all of the above methods compromise accuracy for speed. By contrast, a single-column network [13], which is always synonymous with higher speed and fewer trainable parameters, shows potential for crowd counting.

Under a practical scenario of crowd counting, the size is of equal importance as the accuracy level. Several limits of the platform impose several rigorous restrictions on the time and space complexity of the method: e.g., complex methods may slow down the whole system for crowd counting, and methods that require less memory could be feasible to deploy on contemporary field-programmable gate arrays (FPGAs) or some other edge computing devices.

In this paper, we propose a lightweight CNN network that maintains a good balance between accuracy and size. Our framework follows a single-column style in its backbone and extracts low-level features with reduced spatial resolution in the basic feature extractor (BFE). In particular, skipping connections and bottleneck layers are used in the architecture to increase the efficiency of the feature extractor. Considering the importance of the receptive field size and the size of the network, we replace traditional convolution with à trous convolution, which enlarges the size of the receptive field without reducing spatial resolution in the following layers. One benefit of this strategy is that feature maps in different stages can be integrated without up-sampling from different spatial resolutions. Thus, several à trous convolutional layers form the stacked à trous convolution module (SACM). At the stage of context fusion in the context fusion module (CFM), several short branches connect to different layers. To estimate the density map for crowd counting, the network integrates multi-level information.

Experiments on three main benchmarks, ShanghaiTech [8], UCF_CC_50 [3], and WorldExpo’10 [14], demonstrate the effectiveness of the proposed method. Compared with these methods, the CNN-based network in our method strikes a good balance between size and a series of metrics. This most lightweight network for crowd counting shows attractive performance in terms of feedforward speed and estimated accuracy.

The remainder of the paper is organized as follows. In Section 2, previous works for crowd counting and density maps are reviewed. Section 3 presents the architecture and configuration of our method. In Section 4, experimental results on three main datasets are introduced. Finally, we conclude our work in Section 5.

## 2. Related Work

With the development of deep learning, CNN-based solutions combined with a new technique have shown performance far beyond that of traditional methods. Loy et al. [15] categorized existing traditional crowd counting approaches into: (1) detection-based approaches, (2) regression-based approaches, and (3) density estimation-based approaches. This classification standard is also applicable to approaches based on neural networks.

The vast majority of initial detection-based approaches typically use a sliding window-based solution to detect objects and count the number of people in the vision [16]. Early works on still images were usually performed either in the monolithic style or using parts-based detection [17] by training a classifier (such as support vector machines [18] and random forest [19]) to extract low-level handcrafted features, e.g., Haar wavelets [20] and histograms of oriented gradients [21]. In the fashion of deep-learning-based feature extraction, CNN-based architectures such as Faster-RCNN [22] and YOLO [23] were introduced to tackle the problem. Although these approaches have achieved progress for sparse scenes, poor performance occurs for dense crowds due to hidden objects and background clutter.

In contrast to the complex task of locating all objects in the scene and counting them in detection-based approaches, regression-based approaches attempt to count by regressing a mapping between features extracted from local image patches and their counts [24,25,26]. While these approaches are successful in addressing the issues of occlusion and clutter, most ignore significant spatial location information as they are regressed on the global count.

To tackle the above issues, density maps may be an appropriate expression mode that characterizes the spatial and numerical information of crowds. Lempitsky et al. [27] proposed to learn a linear mapping between local patch features and corresponding object density maps, thereby incorporating spatial details in the learning process. Another solution of learning a non-linear mapping by random forest regression was subsequently proposed by Pham et al [28]. In addition to these methods, a number of approaches based on density maps have been proposed recently [29,30].

Researchers have attempted to use CNN-based approaches to generate density maps or corresponding counts [14,31,32,33] due to the success of CNNs in numerous computer vision tasks. However, due to the limitations of these methods, large-scale variations in crowd scenes may be a notable problem.

Many CNNs based on a multi-column-like architecture (e.g., CrowdNet [34], MCNN [8], and Hydra-CNN [11]) capture a combination of high-level and low-level semantic information to predict the density map for a given scene. To combat the weakness of context fusion, Sam et al. proposed a patch-based architecture called Switch-CNN [12] to exploit local crowd density variation within a scene by sending patches in certain columns, while MoC [35] performs a late selection with a gating CNN in the architecture. CP-CNN [9] goes further by incorporating local and global contextual information from the whole scene image. Sam et al. developed the idea of an expert network group in their new work, and IG-CNN [36] makes the child regressors specialized.

Because optimizing a multi-column network with huge parameters and a complex structure is a different task, the single-column architecture [2,37,38] shows relative advantages. A cascaded multi-task architecture [4] or other effective ideas [1,39,40] improve the performance of the single-column architecture. Counterintuitively, a single-column network with fewer parameters could outperform MCNN on a specific dataset in [13]. Inspired by that result, CSRNet [13], which is powered by the VGG network [41], surpassed the previous state-of-the-art crowd counting solution. Such ideas were considered with pre-trained parameters in [9,12,13], and the methods performed better, but with higher complexity. Kang and Chan [10] applied an image pyramid with an attention map to fuse various scales of objects adaptively. Although its light backbone is attractive, the extra processes of feedforward still slow down their network. Another important idea is to address the issue of inconsistent estimation across different scaled inputs in [42]. Adversarial information, such as adversarial loss and a scale-consistency regularizer, helps the generation network boost density estimation performance.

## 3. Method

Recent works have experimentally introduced new architectures and techniques to challenge previous state-of-the-art works. Most of these efforts have focused on metrics rather than the complexity of their models. For network design, a larger or deeper model is not necessarily better. In other words, the complex engineering problems surrounding us always restrict the application of these methods, and a simple and inexpensive model is almost always easy to deploy.

### 3.1. Architecture

Most proposed CNN-based methods have complicated architectures or a vast number of trainable parameters, which increase the difficulty of model training or make the model feedforward process more time-consuming. For example, some models (e.g., CSRNet [13] and CP-CNN [9]) inherit many parameters from the VGG network [41] for transfer learning. Although these parameters may be frozen during model training, they still dilate the whole model and slow down the speed of this method. Multi-column or multi-branch approaches are synonymous with huge floating-point operations. More attention should be paid to the right-sized architecture. In this paper, the single-column network achieves good performance with less computation and fewer trainable parameters.

Due to the lack of external perspective information about crowds and the presence of multiple scales in an image, it is difficult to estimate crowd counts under a single standard or generate a high-quality density map without spatial distribution information. Conceptually, CNNs need larger receptive fields to recognize the same kind of crowd in a close shot than in a vista. In other words, fusing contextual details or dilating receptive fields is the key factor for a successful crowd counting method. Further-more, context information and multi-scale feature representation play an active part in distinguishing crowd from other objects. Nevertheless, the huge amount of computation cost and parameters restrict the deployment of such applications.

In the following subsections, we design the architecture under the guidance of three key strategies:

- Single-column style
- A minimum sufficient receptive size
- Context embedding

#### 3.1.1. Backbone

In recent years, different types of efficient convolutional neural network architectures for eliminating the redundancy in convolution kernels have been published. SqueezeNet [43] achieves AlexNet-level accuracy on ImageNet with 50× fewer parameters. Another notable work in this direction is the DarkNet Reference network [23], which is optimized for not only parameter count, but also inference speed. MobileNet [44], which uses depth-wise convolution for spatial convolutions, exceeds AlexNet’s performance with only 1.32 million parameters. This idea is extended to pointwise group convolution along with channel shuffling by ShuffleNet [45]. Furthermore, SqueezeNext [46], whose design was guided by considering previous architectures, achieves better classification accuracy with fewer parameters, but avoids using depth-wise-separable convolutions, which are inefficient on some mobile processor platforms. The difference between the performance of these works should not attract too much attention. In a real scenario, better automation capabilities and ease of implementation are more efficient.

Although the above works have studied convolution in-depth, additional complexity may be injected into our experimental comparison. Considering the design of previous works for crowd counting, slightly fine-tuning the architecture composed of traditional convolutional layers may cause impartial competition among such works. Due to our modular design, it is also easy to enhance our model based on the components proposed in the above works for better accuracy or better real-time ability.

**Figure 1:**
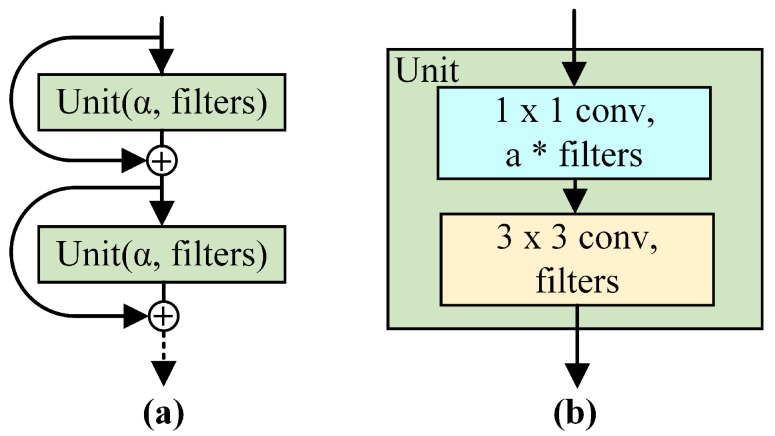
The architecture of the basic unit in our backbone. (**a**) is the layer organization in which a unit is stacked repeatedly and the shortcut connection aids information transfer, and (**b**) is the scheme of the unit, which is composed of a 1×1 bottleneck layer and a 3×3 ordinary convolutional layer. The compression factor (α) and the filters of the Unit (filters) jointly determine the number of filters in each layer.

The design philosophy of the proposed model comes from Tiny Darknet, which is the front-end of the famous object detection network YOLO [23]. As shown in Figure 1, the backbone is repeatedly stacked with a block composed of a 1×1 bottleneck layer and a 3×3 ordinary convolutional layer. In particular, the compression factor (α) and the 1 × 1 filter kernel in the bottleneck layer contribute to limiting the quantity of parameters in this lightweight architecture. In other words, the compression factor (α) and the filters of the unit (filters) jointly determine the number of filters in each layer. A parameter-free shortcut connection is also used to help all information pass through the blocks.

In this paper, the proposed backbone was YOLO style. To further reduce computation, the max-pooling layer was also replaced with a convolutional layer with a stride of 2. However, modern approaches usually compromise spatial resolution to achieve faster inference speed, which leads to poor performance. Due to the importance of the density map resolution and the hard work required to remedy the loss of spatial details, the backbone only reduced the spatial resolution of feature maps twice to obtain the 1/4 feature map. In Table 1, the configuration of the proposed network is presented. The parameters in the convolutional layers are denoted as “(kernel size), (number of filters), (stride), (dilation rate)”. Unless otherwise stated, the stride and dilation rate were all set to 1 by default.

A basic feature extractor (BFE) consisting of three convolutional blocks was employed, which is a fast down-sampling strategy. Following the above extractor, a transition layer was used to compress the depth of the feature maps. This layer provided a “soft landing” to reduce information loss from channel truncation.

Another critical component of our design was the stacked à trous convolution module (SACM). Similar to the component reported in [13], à trous convolution helps the network enlarge the receptive field without extra trainable parameters or any loss of spatial resolution of features. For example, the receptive size of the last layer in the first à trous block {117} is almost three-times larger than the receptive size of the layer above this block {37} in Table 1. In addition, the main idea of à trous convolution is to insert “holes” in basic convolutional kernels, thus enabling employing them easily without other by-products.

Only three à trous convolutional layers with the same dilation rate were deployed in [13], which had the same number of parameters as traditional convolution (same filter size, rate = 1). In contrast to CSRNet, we grouped together three layers as a block with an increasing dilation rate 2, 3, 5 to avoid the “gridding” problem in [47]. At the same time, the receptive sizes of these feature maps increased more efficiently. To ensure an appropriate receptive size of the network, a SACM composed of two consecutive blocks was deployed in this paper.

The final context fusion layer used ReLU activation without batch normalization, while the other convolutional layers were followed with a batch normalization layer and leaky ReLU activation (the slope of the negative section was 0.1).

#### 3.1.2. Context Fusion Module

Several context fusion architectures have been presented: the multi-column architecture was an initial attempt to fuse local context, while CP-CNN [9] and Hydra CNN [11] have further applications. In [10], the classic image pyramid also helped the FCN handle context fusion attributed to multiple scales.

Inspired by the success of spatial pyramid pooling [48], which fuses information of various arbitrary scales, à trous spatial pyramid pooling (ASPP) effectively resamples features at different scales to classify regions of arbitrary scale accurately and efficiently. Various pooling layers (e.g., max pooling) are widely used in current image-to-image models because they can multiply the receptive field in addition to providing other benefits, despite reducing the spatial resolution of feature maps. Attenuation of spatial resolution is a serious problem that leads to loss of information in image-to-image tasks.

In many proposed models [47,49], deconvolution or other upsampling components, which certainly add extra complexity to the original model, are used to recover the resolution. In particular, à trous convolution enlarges the receptive field without loss of spatial details or increasing the number of trainable parameters. This means that CNNs can appear wider with fewer layers, and little attention will be paid to handling feature resolution at different contextual scales.

Multiple parallel à trous convolutional layers with different sampling rates are used in ASPP to generate different scale information in separate branches. The above outputs are joined for final feature fusion. However, the parallel branches in ASSP slow down the inference speed.

In the context fusion module (CFM) we proposed, different contextual features were extracted from different parts of the backbone for further isolated distillation. In other words, various contextual information will not be fused until the last few layers. The experiments in Section 4.2 demonstrate that this design not only improved context fusion, but also reduced the number of parameters and increased the inference speed.

**Figure 2:**
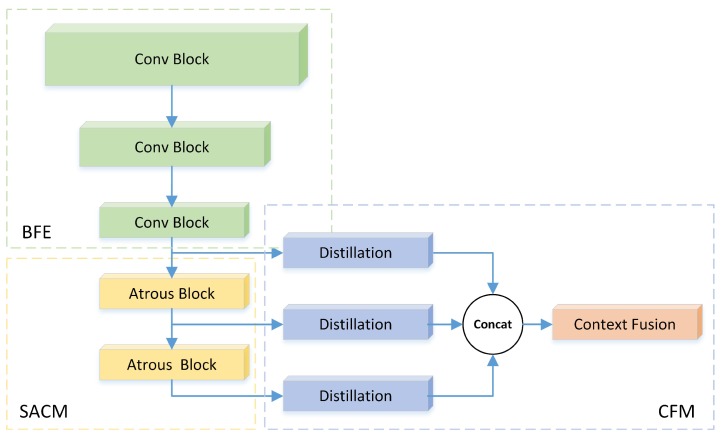
Overview of the proposed network for density estimation. The network extracts common feature maps with reduced spatial resolution using basic feature extractor (BFE). To ensure a diversity of contextual features, the stacked à trous convolution module (SACM) enlarges the gap between the receptive sizes of the feature maps. The context fusion module (CFM) distills different contextual information and fuses it to estimate the density map.

Figure 2 presents the completed scheme of the proposed network for crowd counting. The network extracted common feature maps with reduced spatial resolution using BFE. To ensure the diversity of the contextual features, SACM enlarged the gap between the receptive sizes of feature maps. CFM distilled different contextual information and fused it to estimate the density map.

### 3.2. Training Details

In our proposed method, the Adam optimizer was used to update the parameters of our network with a fixed learning rate during training. The batch size was set to 32, and different fixed learning rates were set for different datasets. For the sake of discussion, the specific setups for different datasets are given in Section 4.3.

#### 3.2.1. Data Augmentation

In the training dataset, we trained our network on images of a single scale. S×S patches (parameter *S* depends on the dataset) were cropped randomly from each image (*N* patches per image), and horizontal flipping generated the other *N* patches; consequently, we obtained 2N patches in total from a single image. If the shorter side of the image was shorter than *S* pixels, the image should be rescaled to fit the above requirement.

#### 3.2.2. Ground Truth

The ground truth in the task of crowd counting was the density map, which demonstrated the total number of the crowd and the spatial distribution information simultaneously. If there was a head at pixel $x_{i}$, we represented it as a delta function $\delta (x-x_{i})$. The ground truth was generated by blurring each head annotation using a normalized Gaussian kernel $G_{\sigma_{i}}\left(x\right)$. Hence, an image with Nheads labeled can be defined as follows:

(1) $$F\left(x\right)=\sum_{i=1}^{N}\delta \left(x-x_{i}\right)*G_{\sigma_{i}}\left(x\right).$$

In the fixed Gaussian kernel, $\sigma_{i}$ is constant for a single image, whose value is defined by the area of each person head. Unfortunately, geometric distortion caused by the perspective effect leads to the variation on the area of the head and limits the quality of the crowd density map.

To tackle the above problem, geometry-adaptive kernels [8] were used to generate the ground truth for highly congested scenes. The parameter $\sigma_{i}$ of the Gaussian kernel was determined by the average distance ${\bar{d}}_{i}$ of the *k* nearest neighbors, which can be represented as:

(2) $$\sigma_{i}=\beta {\bar{d}}_{i}.$$

where β and *k* are hyperparameters. In our experiment, we followed the configuration in [8], where β=0.3 and k=5.

#### 3.2.3. Object Function

The loss function for training the model was the Euclidean distance, which is defined as follows:

(3) $$L\left(\Theta \right)=\frac{1}{N}\sum_{i=1}^{N}{∥F\left(X_{i};\Theta \right)-F_{i}^{GT}∥}_{2}^{2}.$$

where *N* is the size of the training batch and $F(X_{i};\Theta )$ is the density map generated by the proposed model with trainable parameters Θ. L(Θ) denotes the loss between the estimated density map $F(X_{i};\Theta )$ and the ground truth $F_{i}^{GT}$.

## 4. Experimental Results and Discussions

Considering practical applications of our algorithm, we trained and evaluated our network on an Xeon(R) CPU E5-1650 machine with an NVIDIA GeForce GTX 1080 Ti using Tensorflow framework [50]. In addition, the network can run at 28.2 FPS when the image size is resized to 1024×768.

### 4.1. Metrics

The mean absolute error (MAE) and root mean squared error (RMSE) were used to compare between different methods. These metrics are defined as follows:

(4) $$MAE=\frac{1}{N}\sum_{i=1}^{N}\left|C_{i}-C_{i}^{GT}\right|.$$

(5) $$RMSE=\sqrt{\frac{1}{N}\sum_{i=1}^{N}{\left(C_{i}-C_{i}^{GT}\right)}^{2}}.$$

where *N* is the number of images in the evaluation and $C_{i}$ represents the estimated count for the *i*th image, which is the integral value of the corresponding density map. $C_{i}^{GT}$ is the ground truth count.

Because various platforms use different optimization programs, it is difficult to determine which model really has the faster inference speed. In this paper, floating point operations per second (FLOPS) was introduced to measure runtime speed. In particular, only the computation of convolution was considered, and batch normalization, bias adding operation, and other operations were excluded.

### 4.2. Ablation Study and Comparisons

The effectiveness of the configurations of the proposed architecture will be evaluated in this subsection. The ablation study was performed on the ShanghaiTech Part A dataset [8], which is a subset of the ShanghaiTech dataset that includes 482 images of congested scenes and 241,667 annotated heads.

Three configurations are shown in Table 2: A. the completed model; B. the cropped model excluding the context fusion module (CFM); and C. the model that does not compress the depth of feature maps in its bottlenecks (compression rate was set to one). In Table 2, the performance of Config.A is much better than that of Config.B. Obviously, the CFM boosted the performance, which had little impact on calculation. While Config.C was similar to Config.A on both metrics (MAE and MSE), its larger size may slow down the whole model. Consequently, we selected Config.A for further discussion.

Several classic architectures of networks for crowd counting are listed in Table 3. To compare the performance of each method, Table 3 enumerates the number of parameters in the networks, the FLOPS (floating point operations per second) of the main layers, and the two metrics (MAE and RMSE). FLOPS is a de facto standard for computation. In our analysis, the size of the input image for networks was set to 448×448 px. Due to the specific design, multi-column networks (such as MCNN [8], CP-CNN [9], and CrowdNet [34]) are adaptive to scale variations. Although each branch of these networks is short, extra computations are still generated. For example, two networks (MCNN and ours) with similar parameters have completely different computations, such that the FLOPS of MCNN were almost double that of ours. CSRNet [13] is a classic single-column network, but the huge number of parameters from the VGG network slow down the whole method greatly. By contrast, our network had almost 125× fewer parameters than CSRNet. Although many state-of-the-art methods (such as CP-CNN [9], Switching-CNN [12], IG-CNN [36], ACSCP [42], and CSRNet [13]) benefiting from such a transfer learning method perform better, they are very slow for images of high resolution. FCN-7c-3s [10] applies the image pyramid with the attention map to fuse various scales of objects. Its light backbone is attractive, but the feedforward process will run several times per prediction. Consequently, the total FLOPS of FCN-7c-3s were 7× greater than ours. Our network only achieved the second-best performance on MAE and RMSE among the listed networks, but its lowest FLOPS and its lightweight backbone make it more flexible with respect to resource constraints.

**Figure 3:**
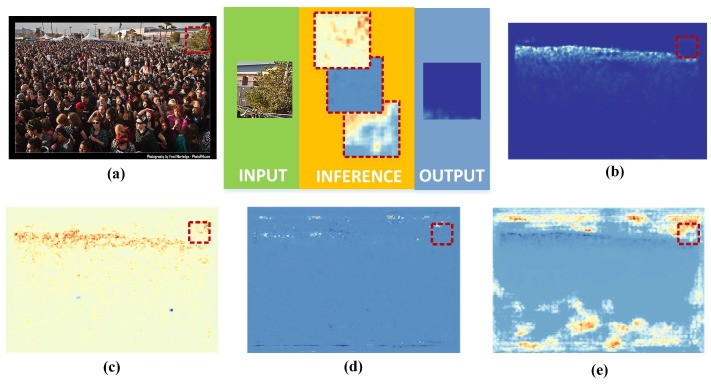
Analysis of the context fusion module (CFM). The outputs of CFM are visualized, and the red bounding boxes mark the same spatial region in different maps. In the feature maps, the color from red to blue reflects the response from strong to weak. (**a**) Input image, (**b**) estimation result, (**c**) first output of CFM, (**d**) second output of CFM, and (**e**) third output of CFM.

Many crowd counting works suffered from semantic mixtures, which make the networks regard other blobs (trees, buildings, and other background) as the crowd. After an analysis of these results, we believe that some kinds of context fusion can aid us in achieving better accuracy. The work in [9] presented the same insight. In Figure 3, the outputs of CFM are visualized, and the red bounding boxes mark the same spatial region in different maps. In these maps, the color from red to blue reflects the response from strong to weak. Although a poor response was observed in some feature maps, the corrected density map was still generated by the other substitutes. The research in [13] showed that the multi-column design performed well against the original intention of the MCNN design for learning different features for each column. In contrast to previous works, CFM can finish homogeneous works in the early stage and then focus on expert specialization works. The component also reduces the amount of computation while ensuring those of different features are learned for each column.

### 4.3. Performance Evaluation

#### 4.3.1. ShanghaiTech Dataset

The ShanghaiTech dataset [8] has two separate subsets: Part A with 482 images (300 images were for training, and the rest were for testing) and Part B with 719 images (400 images were for training, and the rest were for testing). In Part A, images with different resolutions depict highly-congested scenes. By contrast, images with a fixed resolution in Part B were taken from sparse crowd scenes. Following [8], the geometry-adaptive kernel was used to generate ground truth density maps for Part A, and the fixed Gaussian kernel (σ=15) was used for Part B. In this subsection, we train our network on the data augmentation in which 20 patches of 448×448 px are generated from each image. The learning rates in these subsets were $1\times 10^{-3}$ and $1\times 10^{-4}$, respectively.

As shown in Table 4, we evaluated and compared our method with eight other methods on these two subsets separately. In this paper, we further divided all CNN-based methods into two categories: VGG-powered methods and other methods. VGG-powered methods inherited most of their parameters from the VGG network [41], and feature maps generated by extractors consisting of these parameters were used directly in their methods. Although VGG-powered methods have challenged state-of-the-art methods constantly in recent years (such as CP-CNN [9], Switching-CNN [12], IG-CNN [36], ACSCP [42], and CSRNet [13]), we did not compare them with other methods directly due to their huge number of parameters and the benefits of prior knowledge transferred from other tasks. Therefore, the most state-of-the-art results in these two categories are highlighted in bold separately.

**Figure 4:**
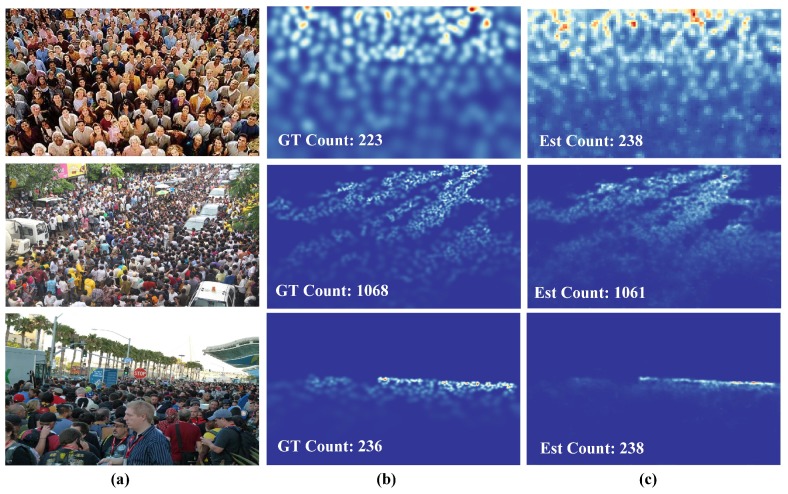
Density estimation results on the ShanghaiTech Part A [8]. (**a**) Input images, (**b**) ground truth density maps, and (**c**) estimation results.

**Figure 5:**
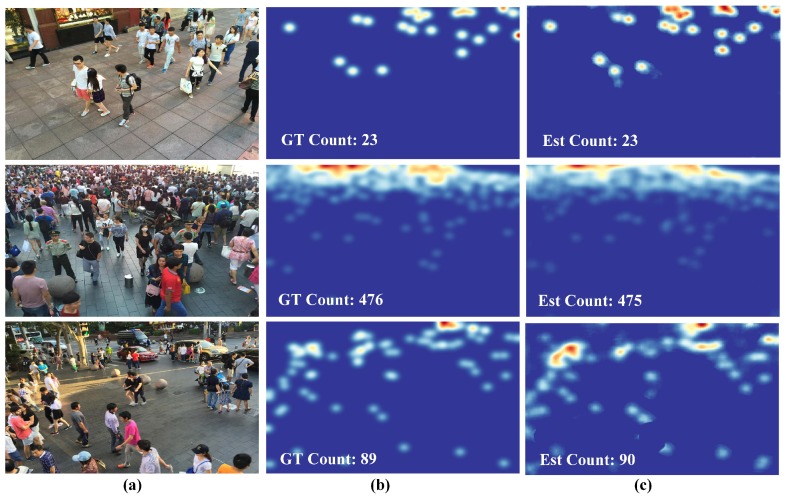
Density estimation results on ShanghaiTech Part B [8]. (**a**) Input images, (**b**) ground truth density maps, and (**c**) estimation results.

Table 4 demonstrates that our method outperformed all methods except VGG-powered methods on ShanghaiTech Part A. On Part B, our method achieved the third-best performance, slightly poorer than the top two. In particular, our network had the fewest parameters and FLOPS: 125× fewer parameters and 40× fewer FLOPS than CSRNet, which achieved the best performance. The proposed network won over FCN-7c-3s by a small margin, but 7× fewer FLOPS relieved the pressure on the computational needs. Density maps generated by the proposed method on the validation dataset are shown in Figure 4 and Figure 5.

#### 4.3.2. UCF_CC_50 Dataset

The UCF_CC_50 dataset [3] contains 50 images of extremely-dense crowds. The dataset exhibits a large variance in crowd counts, with a range of 84–543. Following the standard protocol in [3], a five-fold cross-validation was performed to validate the performance of the proposed method.

**Figure 6:**
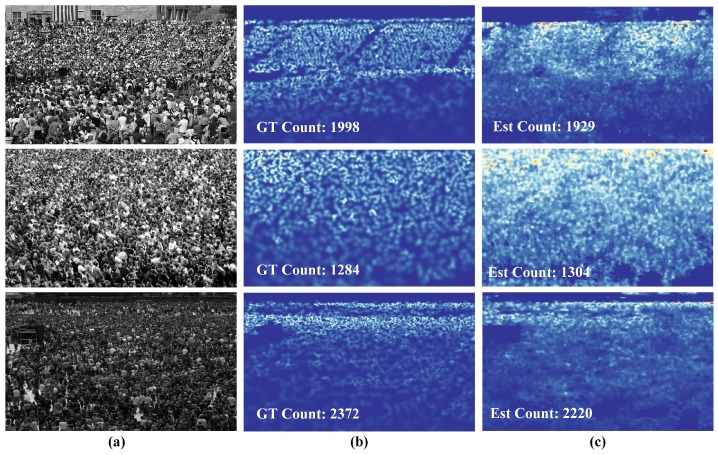
Density estimation results on the UCF_CC_50 [3]. (**a**) Input images, (**b**) ground truth density maps, and (**c**) estimation results.

The geometry-adaptive kernel was also performed in these highly-congested scenes for ground-truth density map generation. In consideration of the specific properties of this dataset, 448×448 patches were cropped, and a total of 60 samples was generated from each image. During the training time, the learning rate was set to $1\times 10^{-3}$. The results are listed in Table 5, which demonstrated the improved performance of our method. Our network achieved comparable MAE and RMSE scores, with current state-of-the-art MAE values, but high RMSE values. These results demonstrated that our model had low bias and high variance, which means that the model was not robust enough for this dataset. Despite this quality, fewer parameters and the least FLOPS among the top four methods make our method more flexible for practical implementation. Figure 6 illustrates the density maps obtained using the proposed method.

#### 4.3.3. The WorldExpo’10 Dataset

The WorldExpo’10 dataset, which was first introduced by Zhang et al. [14], includes 3980 annotated frames from 1132 annotated video sequences captured by 108 surveillance cameras. All frames in the dataset were divided into two sets: the training set contained 3380 frames, and the testing set had 600 frames from five different scenes. The region of interest (ROI) was provided for each scene. Following the evaluation protocol mentioned in [14], a human-like Gaussian kernel was adopted, and only the crowd in the ROI was taken into consideration.

**Figure 7:**
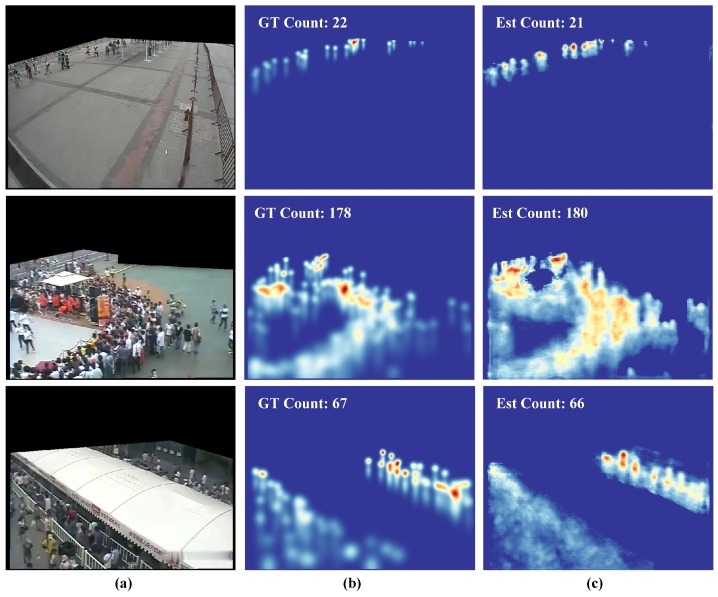
Density estimation results on the WorldExpo’10 dataset [14]. (**a**) Input images, (**b**) ground truth density maps, and (**c**) estimation results.

In this subsection, our network was trained on the data augmentation in which 40 patches of 448×448 px were generated from each image. Considering five different crowd scenes in this dataset, the learning rate was set from $1\times 10^{-3}$ to $1\times 10^{-5}$ in the specific scene (from the crowded scene to the sparse scene). The results are listed in Table 6. The average performance (Avg. MAE) of VGG-powered models was still dominant. In particular, our model achieved top rankings and even surpassed VGG-powered models in several scenes. In Sce.2, more complex models (such as CP-CNN and Switching-CNN) achieved better performance, while this scene was the most complex scene among the five scenes. The results of these experiments demonstrated that the complexity of the model should be adapted to the specific task. Figure 7 illustrates the density maps obtained using the proposed method.

## 5. Conclusions

In this paper, we proposed a lightweight CNN-based crowd counting network for better performance in crowd counting. Accuracy and complexity have gradually become unbalanced in previous research. Benefiting from a specially-designed BFE, SACM, and CFM, the proposed network not only achieved excellent performance with the three main datasets, but also was the smallest network among the recent series reported in the literature. Although VGG-powered networks had better rankings, the smaller size and shorter feedforward time made our method more favorable.

Future work will cover the extension of the proposed method to more robust performance, as well as the deployment of our method with realistic scenes. The performance on different platforms will also be investigated. Finally, domain adaption problems caused by limited expensive datasets will be further studied.

## Figures and Tables

**Table 1:** Configuration of the proposed fully-convolutional network. The parameters in the convolutional layers are denoted as “(kernel size), (number of filters), (stride), (dilation rate)”. Unless otherwise stated, the stride and dilation rate are all set to 1 by default.

Layer Name	Output Size	Configuration
Conv Block 1	448×448	3×3,16
Conv Block 2	224×224	3×3,32,stride2
		$\left[\begin{array}{c} \begin{array}{c} 1\times 1,16\\ 3\times 3,32 \end{array} \end{array}\right]$
Conv Block 3	112×112	3×3,64,stride2
		$\left[\begin{array}{c} \begin{array}{c} 1\times 1,32\\ 3\times 3,64 \end{array} \end{array}\right]\times 2$
Transition	112×112	$\left[\begin{array}{c} \begin{array}{c} 1\times 1,32\\ 3\times 3,32 \end{array} \end{array}\right]$
Atrous Block 1	112×112	$\left[\begin{array}{c} \begin{array}{c} 1\times 1,16\\ 3\times 3,32,rate=2 \end{array} \end{array}\right]$
		$\left[\begin{array}{c} \begin{array}{c} 1\times 1,16\\ 3\times 3,32,rate=3 \end{array} \end{array}\right]$
		$\left[\begin{array}{c} \begin{array}{c} 1\times 1,16\\ 3\times 3,32,rate=5 \end{array} \end{array}\right]$
Atrous Block 2	112×112	$\left[\begin{array}{c} \begin{array}{c} 1\times 1,16\\ 3\times 3,32,rate=2 \end{array} \end{array}\right]$
		$\left[\begin{array}{c} \begin{array}{c} 1\times 1,16\\ 3\times 3,32,rate=3 \end{array} \end{array}\right]$
		$\left[\begin{array}{c} \begin{array}{c} 1\times 1,16\\ 3\times 3,32,rate=5 \end{array} \end{array}\right]$
Distillation	112×112	$\left[\begin{array}{c} \begin{array}{c} 1\times 1,16\\ 3\times 3,32 \end{array} \end{array}\right]$
		1×1,1
Context Fusion	112×112	1×1,1

**Table 2:** Comparison of the proposed networks with different configurations on the ShanghaiTech Part A dataset. Config., configuration.

Methods	Parameters	FLOPS	MAE	RMSE
Config.A	**130.40 k**	**2040 m**	78.5	**126.4**
Config.B	119.60 k	1911 m	92.1	142.0
Config.C	223.46 k	3389 m	**78.1**	129.2

**Table 3:** Comparison of the networks on the ShanghaiTech Part A dataset.

Methods	Parameters	FLOPS	MAE	RMSE
MCNN [8]	127.95 k	5384 m	110.2	185.9
CSRNet [13]	16.26 m	82,889 m	**68.2**	**115.0**
FCN-7c-3s [10]	150.92 k	14,327 m	80.6	126.7
Ours	**130.40 k**	**2040 m**	78.5	126.4

**Table 4:** Comparison of networks on the ShanghaiTech dataset.

	Part A		Part B	
**Method**	**MAE**	**RMSE**	**MAE**	**RMSE**
MCNN [8]	110.2	173.2	26.4	41.3
Cascaded-MTL [4]	101.3	152.4	20.0	31.1
FCN-7c-3s [10]	80.6	126.7	**10.2**	**18.3**
Ours	**78.5**	**126.4**	12.8	22.1
Switching-CNN [12]	90.4	135.0	21.6	33.4
CP-CNN [9]	73.6	106.4	20.1	30.1
CSRNet [13]	**68.2**	115.0	**10.6**	**16.0**
IG-CNN [36]	72.5	118.2	13.6	21.1
ACSCP [42]	75.7	**102.7**	17.2	27.4

**Table 5:** Comparison of networks on the UCF_CC_50 dataset.

Method	MAE	RMSE
Onoro et al. [11]	465.7	371.8
MCNN [8]	377.6	509.1
Cascaded-MTL [4]	322.8	**341.4**
Ours	**299.1**	391.8
Switching-CNN [12]	318.1	439.2
CP-CNN [9]	295.8	**320.9**
IG-CNN [36]	291.4	349.4
ACSCP [42]	**291.0**	404.6

**Table 6:** Comparison of the networks on the WorldExpo’10 dataset.

Method	Sce.1	Sce.2	Sce.3	Sce.4	Sce.5	Avg.
Zhang et al. [14]	9.8	**14.1**	14.3	22.2	3.7	12.9
MCNN [8]	3.4	20.6	12.9	13.0	8.1	11.6
FCN-7c-3s [10]	2.5	16.5	12.2	20.5	**2.9**	10.9
Ours	**2.4**	27.0	**7.9**	**8.4**	3.1	**9.76**
Switching-CNN [12]	4.2	14.9	14.2	18.7	4.3	11.2
CP-CNN [9]	2.9	14.7	10.5	10.4	5.8	8.9
CSRNet [13]	2.9	**11.5**	**8.6**	16.6	3.4	8.6
IG-CNN [36]	**2.6**	16.1	10.15	20.2	7.6	11.3
ACSCP [42]	2.8	14.05	9.6	**8.1**	**2.9**	**7.5**
